# Supplementary material for: Hybrid Models and Biological Model Reduction with PyDSTool
Source: PLoS Comput Biol. 2012 Aug 9;8(8):e1002628. doi: 10.1371/journal.pcbi.1002628 (PMC3415397; doi:10.1371/journal.pcbi.1002628)
Supplement: Text S4 — Complete source code for the PyDSTool package (version 0.88.120504). Includes API documentation and help files linking to web pages. This file is identical to the current public release on Sourceforge.net. (ZIP) [file pcbi.1002628.s004.zip › PyDSTool/html/toc-PyDSTool.Toolbox.optimizers.line_search-module.html]

xml version="1.0" encoding="ascii"?


line\_search


# Module line\_search

---

## Classes

AdaptiveLastStepModifier  
 BacktrackingSearch  
 CubicInterpolationSearch  
 DampedLineSearch  
 FibonacciSectionSearch  
 FixedLastStepModifier  
 GoldenSectionSearch  
 GoldsteinRule  
 HyperbolicLineSearch  
 QuadraticInterpolationSearch  
 ScaledLineSearch  
 SimpleLineSearch  
 StrongWolfePowellRule  
 WolfePowellRule  

## Variables

line\_search\_\_all\_\_

---

[hide private]
